# Spheroids of Bladder Smooth Muscle Cells for Bladder Tissue Engineering

**DOI:** 10.1155/2021/9391575

**Published:** 2021-11-11

**Authors:** Tim Gerwinn, Souzan Salemi, Lisa Krattiger, Daniel Eberli, Maya Horst

**Affiliations:** ^1^Department of Urology, University Children's Hospital Zurich, Zurich CH-8032, Switzerland; ^2^Children's Research Center, University Children's Hospital Zurich, Zurich CH-8032, Switzerland; ^3^Laboratory for Urologic Oncology and Stem Cell Therapy, Department of Urology, University Hospital Zurich, Zurich CH-8091, Switzerland; ^4^Department of Obstetrics, University Hospital Zurich, Zurich CH-8091, Switzerland

## Abstract

Cell-based tissue engineering (TE) has been proposed to improve treatment outcomes in end-stage bladder disease, but TE approaches with 2D smooth muscle cell (SMC) culture have so far been unsuccessful. Here, we report the development of primary bladder-derived 3D SMC spheroids that outperform 2D SMC cultures in differentiation, maturation, and extracellular matrix (ECM) production. Bladder SMC spheroids were compared with 2D cultures using live-dead staining, qRT-PCR, immunofluorescence, and immunoblotting to investigate culture conditions, contractile phenotype, and ECM deposition. The SMC spheroids were viable for up to 14 days and differentiated rather than proliferating. Spheroids predominantly expressed the late myogenic differentiation marker MyH11, whereas 2D SMC expressed more of the general SMC differentiation marker *α*-SMA and less MyH11. Furthermore, the expression of bladder wall-specific ECM proteins in SMC spheroids was markedly higher. This first establishment and analysis of primary bladder SMC spheroids are particularly promising for TE because differentiated SMCs and ECM deposition are a prerequisite to building a functional bladder wall substitute. We were able to confirm that SMC spheroids are promising building blocks for studying detrusor regeneration in detail and may provide improved function and regenerative potential, contributing to taking bladder TE a significant step forward.

## 1. Introduction

End-stage bladder disease (EBD) in children is mainly caused by spina bifida, posterior urethral valves, and other congenital malformations. EBD can lead to severely reduced bladder capacity and loss of compliance [1]. This results in increased intravesical pressure, secondary vesicoureteral reflux, urinary tract infections, and incontinence. If left untreated, EBD leads to kidney damage and even chronic kidney disease. Augmentation cystoplasty using intestinal segments represents the gold standard therapy for children with EBD after conservative therapy is exhausted [2, 3]. Current treatment options offer limited success and high morbidity [4], so research interest has recently focused on cell-based tissue engineering (TE) to improve treatment outcomes and reduce complications.

Unfortunately, the first human bladder TE trials failed to deliver satisfactory results. After initial success in research [5], larger clinical trials showed insufficient development of bladder capacity and compliance due to fibrosis of the engineered bladder wall substitute [6]. Successful bladder TE requires the reliable isolation and expansion of smooth muscle cells (SMCs) *in vitro* and the preservation of the SMC-specific phenotype while avoiding undesired developments such as cellular senescence, dedifferentiation, and transdifferentiation [7]. Even mature SMCs derived from healthy tissue have limited proliferative capacity and lose contractile phenotype during *in vitro* 2D cell culture expansion [8]. Another difficulty is the fact that bioengineered tissue must be produced using the patient's own functionally impaired cells to prevent inflammatory response against allogeneic donor cells. To address this issue, our group has most recently investigated improving SMC function through coculture with predifferentiated smooth muscle like adipose-derived stem cells [9, 10]. Further obstacles when trying to engineer functional bladder wall substitutes include the lack of contractibility, unsatisfactory urine barrier, insufficient vascularization, and innervation, all of which lead to impaired wound healing and tissue fibrosis [11].

Spheroid cultures may offer a way around these problems. A spheroid is a 3D cell aggregate established from a single primary cell type or from a multicellular mixture of primary cells, immortalized cell lines, or fragments of human tissue [12]. Spheroids are already widely used in cancer and drug delivery research [13–15]. A wide range of spheroid fabrication techniques have been established [16, 17]. What they have in common is their similarity to embryonic development, during which cells aggregate without any external influence. The generation of 3D cell cultures relies on the common basic principle of self-assembly, because cells cannot attach to a biomaterial surface and are therefore forced to interact with each other [18, 19]. This close interaction results in increased cell–cell and cell–extracellular matrix (ECM) interaction. [15]. In contrast to conventional 2D monolayer cultures, these 3D cultures exhibit higher similarity to real tissue. Therefore, spheroid cultures exhibit several unique properties that make them ideal for TE applications. Spheroids can be generated in defined sizes and in large amounts by high-throughput fabrication. They exhibit improved regenerative properties compared to 2D single cell approaches and allow the generation of more complex tissues by coculturing multiple cell types in one spheroid [20]. Moreover, their function can be improved by *in vitro* preconditioning under different culture conditions [21]. Spheroids even offer the potential to fuse into macrotissue constructs for large-scale TE [20, 22]. In the last few years, spheroid cultures have become increasingly important in the field of TE because of their remarkable regenerative properties. The cells within spheroids have an improved differentiation potential and are more resistant against hypoxia and apoptotic cell death [21, 23]. Moreover, they produce higher amounts of growth factors when compared to 2D cultures [23, 24].

There is available literature on various urologic 3D culture models for cancer cell lines or urothelial cells. These *in vitro* models are used in the characterization, diagnosis, and treatment of bladder cancer and urinary tract infections [15]. However, little attention has been paid to the detrusor muscle, which consists of SMCs and is crucial for successful and functional bladder TE.

We hypothesize that SMC spheroids' improved regenerative properties and increased ECM deposition provide superior building blocks for bladder TE. This study is aimed at establishing and characterizing SMC spheroids for future spheroid-based bladder TE projects.

## 2. Material and Methods

### 2.1. SMC Isolation from Rat Bladder Biopsies

Male Wistar rat bladders were harvested and washed in phosphate-buffered saline (PBS, Invitrogen), containing 1% penicillin/streptomycin (PS, Invitrogen) and according to published protocols [10, 25]. The urothelial layer was scraped off with a scalpel. The detrusor muscle was minced into 1 × 1 mm pieces, placed on 10 cm culture dishes, and left to dry for 5 min. All animal work was in line with local research ethics and animal welfare. Cells were cultured in cell culture medium containing DMEM/F12+GlutaMAX (Invitrogen), 10% fetal bovine serum (FBS, Merck), 1% PS, 12.5 *μ*g/500 mL fibroblast growth factor, and 5 *μ*g/500 mL epidermal growth factor, and 5 mg/500 mL human insulin was added [26]. Culture dishes were incubated at 37°C in a humidified atmosphere with 5% CO_2_. Medium was changed every 3–4 days. Remaining tissue pieces were removed after one week, and SMCs were expanded up to passage 5.

### 2.2. SMC Spheroid Production

We used the Sphericalplate 5D® (Kugelmeiers, Erlenbach, Zurich, Switzerland). Briefly, the plate consists of 24 wells, of which 12 wells are manufactured with 750 punched-out nonadhesive microwells, each shaped as an inverted pyramid, in which the SMCs accumulated to form the spheroids. This allows 750 spheroids to be generated in one well. The SMCs were seeded in 2 mL of culture medium with the desired cell concentration. The cell suspension was thoroughly mixed inside each well to ensure even distribution of the SMCs in the microwells and thus obtain spheroids of similar size. Quantities of 500, 1000, and 1500 cells were seeded per spheroid. Medium was changed every 3 days.

### 2.3. Monitoring of SMC Spheroid Formation

SMC spheroids were imaged daily during the first week of culture using an inverted microscope (Leica CTR 6000). The surface area of 12 representative spheroids per cell number condition was measured daily with the ImageJ Software (NIH, USA). Each experimental seeding condition was monitored in triplicate.

### 2.4. Live-Dead Staining Study

SMC viability for spheroids and 2D was evaluated qualitatively on days 2, 4, and 7 by calcein-AM and propidium iodide (PI) dual staining. Spheroids were subsequently evaluated every second day up to week 3 of spheroid culture. Briefly, culture medium was carefully removed without disturbing the spheroids inside the Sphericalplate 5D®. Then, 1 mL of fresh culture medium containing 1 *μ*L calcein-AM (10 *μ*g/*μ*L) (Invitrogen), 1 *μ*L propidium iodide (10 *μ*g/*μ*L) (Sigma), and 1 *μ*L Hoechst (10 *μ*g/*μ*l) (Thermo Fisher) was added to the spheroids. After incubation at 37°C and 5% CO_2_ for 30 minutes, the supernatant was carefully removed, and the spheroids were harvested and transferred to a 1.5 mL Eppendorf tube. Spheroids were washed twice with culture medium and resuspended with fresh culture medium. The spheroids were immediately imaged on a microscope slide with a fluorescence microscope (Leica CTR 6000).

### 2.5. Quantitative Real-Time PCR

SV Total RNA Isolation System kit (Promega) was used according to the manufacturer's protocol [27]. RNA was measured and reverse-transcribed with random primers (High-Capacity cDNA reverse transcription, Applied Biosystems). Primers for smoothelin (Rn01453095_m1), calponin (Rn00582058_m1), and MyH11 (Rn01530326_m1) were acquired from Applied Biosystems. Collagen I (Rn01526722_m1), collagen III (Rn01437681_m1), elastin (Rn01499782_m1), and fibronectin (Rn00569575_m1) were purchased from Thermo Fisher. GAPDH (Rn01775763-g1) was used to normalize cDNA concentrations. The data were analyzed by measuring the cycle threshold (CT) values, which were between 20 and 30 in all the samples. Results are shown as means ± standard deviation; each sample was measured in triplicate. We compared the fold increase in gene expression from early-stage spheroids harvested on days 2 and 4 of culture to spheroids kept in culture for 7 days. Genes in 2D SMC were analyzed and presented in the same way.

### 2.6. Immunofluorescent Staining

For 2D SMCs, cells were cultured on Lab-Tek chamber slides (Thermo Scientific). The indirect immunostainings for both spheroids and 2D SMCs were performed on day 2 of culture. Samples were stained at 4°C overnight using the following primary antibodies for the contractile SMC proteins: anti-*α*-smooth muscle actin (*α*-SMA) (Sigma, A5228, 1 : 200), anti-calponin (Sigma, C2687, 1 : 100), anti-smoothelin (Novus, NBP2-37931, 1 : 100), and anti-myosin heavy chain 11 (MyH11) (Santa Cruz, SC-6956, 1 : 50). The slides were incubated with a Cy3-conjugated secondary antibody (Sigma, 1 : 500) at room temperature for 1 h.

For ECM proteins, anti-collagen I (Abcam, ab260043, 1 : 150), anti-collagen III (Abcam, ab7778, 1 : 150), anti-fibronectin (Santa Cruz, sc-59826, 1 : 300), and anti-elastin (Abcam, ab9519, 1 : 100) were used. Samples were stained at 37°C for 4 h. The slides were incubated with a FITC-conjugated secondary antibody (Sigma, 1 : 500) at 4°C overnight. The slides were counterstained with DAPI (4′,6-diamidino-2-phenylindole, Sigma, 1 : 400) and analyzed with an inverted confocal microscope (Leica DMI6000 AFC, Model SP8). For negative controls, the primary antibody was omitted. The specificity of the commercial antibodies had been thoroughly validated in our previous studies [28, 29].

### 2.7. Immunoblotting

The total protein was measured with the BCA Protein Assay Kit (Thermo Scientific). Briefly, 1.5 mg/ml of protein was used for the WES (automated western blotting, Protein Simple WES) sample preparation with 12–230 kDa cartridge kit, and the proteins were separated in WES with a capillary cartridge according to the manufacturer's protocols (Protein Simple WES, Germany). Mouse primary antibodies were used against *α*-SMA (Novus, NBP2-33006, 1 : 100), calponin (Sigma-Aldrich, C2687, 1 : 400), MyH11 (Santa Cruz, sc-59826, 1 : 10), and smoothelin (Novus, NBP2-37931, 1 : 100). Mouse anti-GAPDH (Novus, NB300-221, 1 : 100) served as internal control. Protein expression was normalized and quantified in relation to GAPDH and analyzed using the Compass software (Protein Simple). Protein expression on day 2 and day 4 was compared to that on day 7.

### 2.8. Statistics

Results are presented as mean and standard error of the mean (SEM). Statistical analysis was performed using GraphPad Prism 8 (GraphPad Software, La Jolla, California) by one-way ANOVA and Bonferroni comparison. Statistically significant variances were defined as ^∗^*p* < 0.05, ^∗∗^*p* < 0.01, ^∗∗∗^*p* < 0.001, and ^∗∗∗∗^*p* < 0 .0001. All experiments were performed in triplicate.

## 3. Results

### 3.1. Smooth Muscle Cell Spheroid Formation

**Figure 1:**
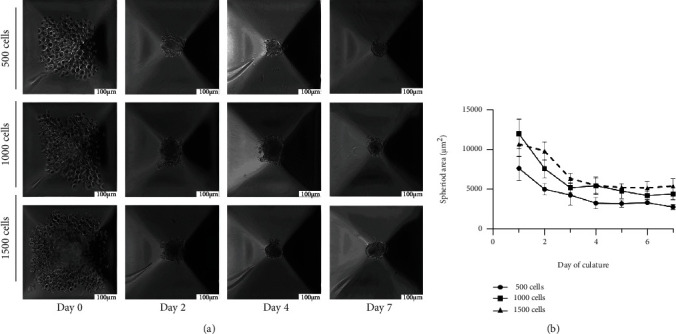
SMC spheroid formation and size development over time. (a) Spheroid formation in various cell number conditions per spheroid was observed daily for 1 week, and mean transverse cross-section area was plotted. All conditions form spheroids equally quickly. (b) Seeded cell number and spheroid size correlated as 1500 cells/spheroid yield the largest transverse cross-section area. Scale bar is 100 *μ*m.

To explore differences in SMC spheroid formation potential and cultural behavior, we seeded and cultured the cells in groups of 500, 1000, and 1500 cells for 2, 4, and 7 days.  Figure 1(a) shows the formation of SMC spheroids with various seeding numbers over a one-week observation period. The SMCs sedimented to the bottom of the microwells within the first 24 hours of culture. All seeded conditions formed spheroids, and there was no noticeable difference in the time required for spheroid formation between the cell number conditions. We evaluated the relationship between the initial number of seeded cells and the resulting spheroid diameter as measured from photographs of the spheroids in culture taken at various time points (Figure 1(b)). The highest cell number produced the largest spheroids. On day 2, the transverse cross-sectional area for 1500 cells per spheroid (9871 *μ*m^2^ ± 215 *μ*m^2^, mean ± SEM) was larger than that for 500 cells (5103 ± 314, *p* < 0.001) and 1000 cells (7668 ± 682, *p* < 0.05). On day 4, the transverse cross-sectional area for 1500 cells (5565 ± 100) was larger than that for 500 cells (3373 ± 275, *p* < 0.001) and larger than that for 1000 cells (5546 ± 233). On day 7, the transverse cross-sectional area for 1500 cells (5505 ± 320) was larger than that for 500 cells (2891 ± 238, *p* < 0.001) and for 1000 cells (4515 ± 51, *p* < 0.05).

### 3.2. Live-Dead Staining Study

**Figure 2:**
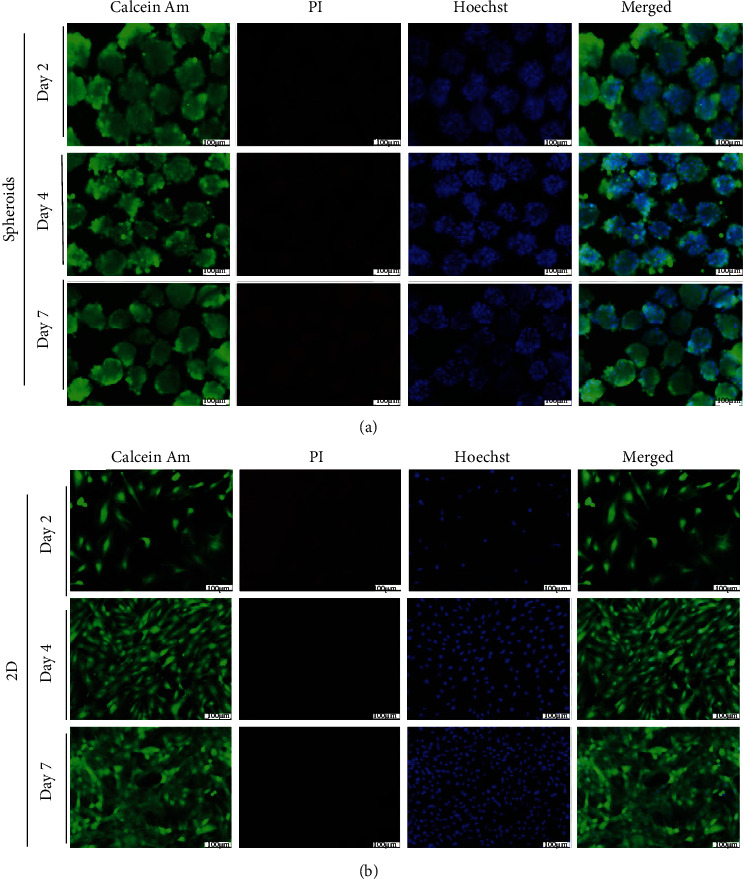
Live-dead staining study. Live cells stained green with calcein-AM, dead cells stained red with propidium iodide (PI), and nuclei stained blue with Hoechst. (a) SMC spheroids remain live until day 7 of culture. (b) SMCs in 2D as control show no dead cells until day 7 of culture. Scale bar is 100 *μ*m.

For all subsequent experiments, we proceeded with the most efficient cell seeding number of 1500 cells/spheroid, the highest cell number condition tested. Cells were examined microscopically using calcein-AM, PI, and Hoechst staining. We found high viability of the SMCs in spheroids with few dead cells after 7 days. Both spheroids and 2D gave similar results during the first week of culture (Figure 2). Furthermore, we continued to evaluate the viability of the SMC spheroids until day 21 of culture (Figure S2). Small central necrosis zones within the SMC spheroids started developing at day 10. The necrosis zones were clearly visible and increased in size from 2 weeks onwards. This was accompanied by decreasing Hoechst uptake of the nuclei, which indicates cell necrosis.

### 3.3. Contractile and ECM Gene Expression in Spheroids Compared to 2D

**Figure 3:**
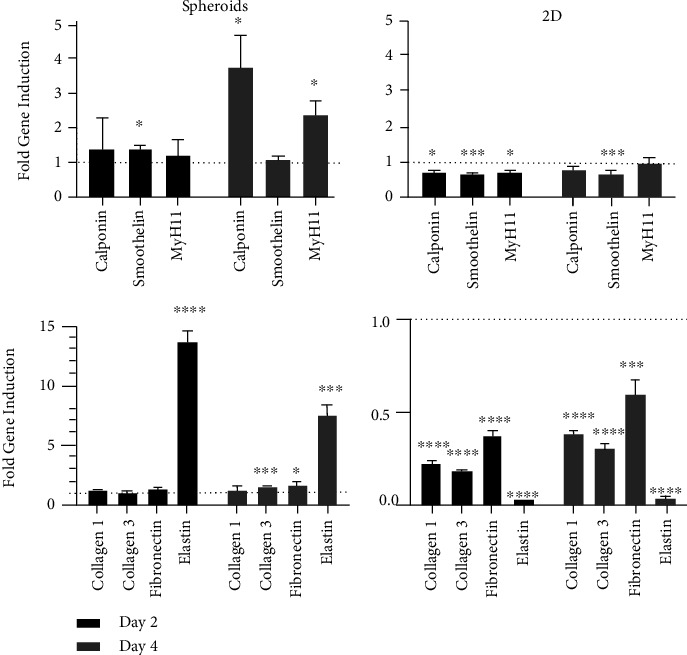
Quantitative real-time PCR. SMC-specific contractile gene induction for calponin, smoothelin, and MyH11 is increased in early spheroids, particularly on day 4 of spheroid culture. Interestingly, comparison shows a stark contrast, with almost no changes in 2D SMC gene induction over time.

To quantify contractile and ECM gene expression in spheroids and 2D SMCs, qRT-PCR was performed (Figure 3). The expression levels of contractile markers calponin (day 2: 1.40 ± 0.87, mean ± SEM and day 4: 3.71 ± 0.53, *p* < 0.05), smoothelin (day 2: 1.37 ± 0.1, *p* < 0.05 and day 4: 1.1 ± 0.3), and MyH11 (day 2: 1.2 ± 0.44 and day 4: 2.4 ± 0.25, *p* < 0.05) were higher in early spheroids than in spheroids on day 7. ECM gene expression in spheroids presented a similar pattern, with greater induction on days 2 and 4 than on day 7: collagen I (day 2: 1.28 ± 0.09 and day 4: 1.25 ± 0.05), collagen III (day 2: 1.01 ± 0.04 and day 4: 1.53 ± 0.05, *p* < 0.001), fibronectin (day 2: 1.36 ± 0.01 and day 4: 1.62 ± 0.18, *p* < 0.05), and elastin (day 2: 13.63 ± 0.90, *p* < 0.0001 and day 4: 7.39 ± 0.56, *p* < 0.001).

In contrast, contractile gene expression for 2D SMC displayed increasing induction of calponin (day 2: 0.48 ± 0.2, *p* < 0.05 and day 4: 0.8 ± 0.05), smoothelin (day 2: 0.69 ± 0.1, *p* < 0.001 and day 4: 0.66 ± 0.05, *p* < 0.001), and MyH11 (day 2: 0.73 ± 0.04, *p* < 0.05 and day 4: 0.97 ± 0.09) with increasing culturing duration. The same pattern was observed for the ECM gene expression, which showed a significant increase in expression with increase in culture duration.

The results showed increases over time in collagen I (day 2: 0.22 ± 0.01, *p* < 0.0001 and day 4: 0.38 ± 0.01, *p* < 0.0001), collagen III (day 2: 0.18 ± 0.01, *p* < 0.0001 and day 4: 0.3 ± 0.02, *p* < 0.0001), fibronectin (day 2: 0.37 ± 0.03, *p* < 0.0001 and day 4: 0.6 ± 0.04, *p* < 0.001), and elastin (day 2: 0.02 ± 0.0, *p* < 0.0001 and day 4: 0.03 ± 0.01, *p* < 0.0001).

Gene induction of ECM genes in early spheroids is higher; elastin in particular showed a significantly higher gene induction than day 7 of culture. 2D SMCs show a significantly lower ECM gene expression in early culture conditions. The dotted line represents the gene induction on day 7 of culture, which served as reference to calculate the fold gene induction. ^∗^*p* < 0.05, ^∗∗^*p* < 0.01, ^∗∗∗^*p* < 0.001, and ^∗∗∗∗^*p* < 0.0001.

### 3.4. Contractile and ECM Protein Expression in Spheroids Compared to 2D

**Figure 4:**
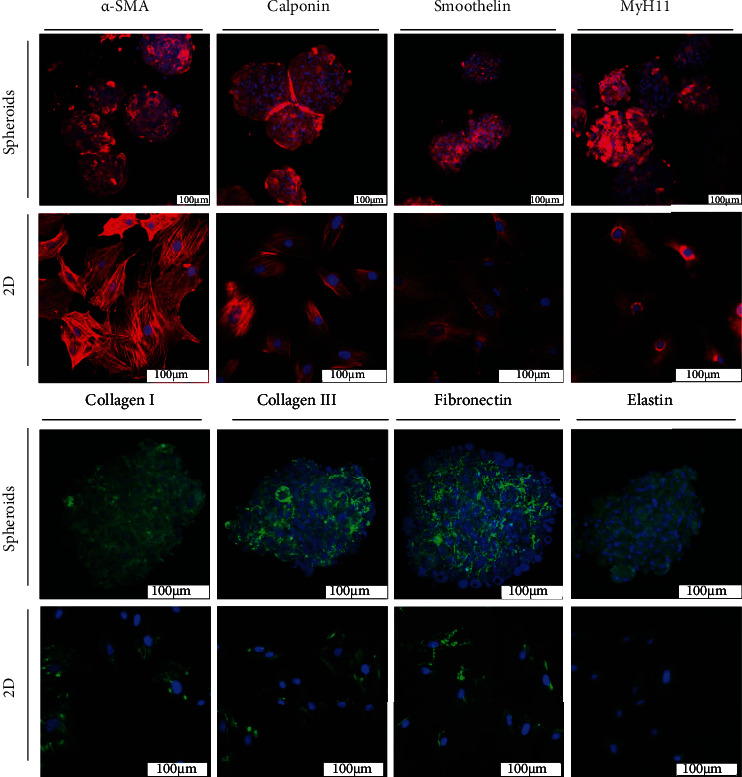
Immunofluorescent staining. SMC spheroids and 2D (monolayer) SMCs stained positive for SMC lineage-specific markers *α*-smooth muscle actin, calponin, smoothelin, and myosin heavy chain 11. Bladder wall ECM proteins collagen I, collagen III, elastin, and fibronectin are more strongly expressed in SMC spheroids. Nuclei stained blue with DAPI. Scale bar is 100 *μ*m.

To investigate the contractile protein expression of SMCs in spheroids and 2D, cells were stained on day 2 of culture and visualized by confocal microscopy. Pictures presented in Figure 4 display expression of all SMC-specific contractile protein markers—*α*-SMA, calponin, smoothelin, and MyH11—in both SMC spheroid and 2D monolayer cultures. However, the ECM protein expression for collagen I, collagen III, elastin, and fibronectin was apparently more pronounced in SMC spheroids than in 2D SMCs.

**Figure 5:**
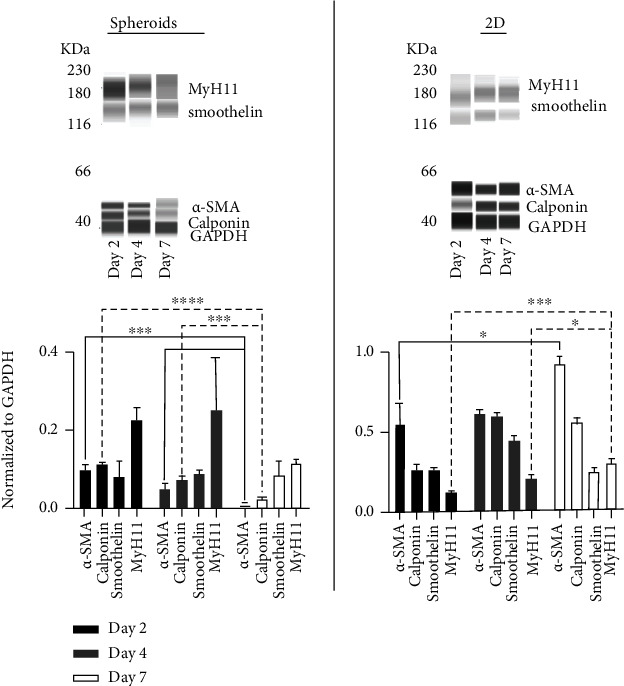
Immunoblotting. The figure displays summarized representative bands and absolute protein concentrations normalized to GAPDH. Both SMC spheroids and 2D SMCs express the characteristic contractile marker proteins (*α*-SMA, calponin, smoothelin, and MyH11). Immunoblotting indicates that SMC spheroids quickly shift to a more differentiated phenotype than do 2D SMCs. ^∗^*p* < 0.05, ^∗∗^*p* < 0.01, ^∗∗∗^*p* < 0.001, and ^∗∗∗∗^*p* < 0.0001. See figure S5 for representative bands for all time points.

Immunoblotting was performed to quantify contractile protein in spheroids and 2D SMCs. In line with gene expression and staining results, immunoblotting showed expression of all characteristic SMC contractile-related proteins *α*-SMA, calponin, smoothelin, and MyH11 in spheroids and 2D SMCs (Figure 5). Early spheroids on day 2 and day 4 expressed higher quantities of contractile proteins *α*-SMA (day 2: 0.10 ± 0.01, mean ± SEM, *p* < 0.001; day 4: 0.05 ± 0.01, *p* < 0.05; and day 7: 0.008 ± 0.002), calponin (day 2: 0.12 ± 0.002, *p* < 0 .0001; day 4: 0.08 ± 0.01, *p* < 0.001; and day 7: 0.02 ± 0.004), and MyH11 (day 2: 0.23 ± 0.03, day 4: 0.25 ± 0.13, and day 7: 0.12 ± 0.005) than on day 7 of culture. However, no changes were observed in smoothelin protein expression over time (day 2: 0.08 ± 0.04, day 4: 0.09 ± 0.01, and day 7: 0.09 ± 0.02).

Consistent with the 2D SMC gene induction, an increase in protein expression over time was indicated for *α*-SMA (day 2: 0.54 ± 0.14, *p* < 0.05; day 4: 0.61 ± 0.03; and day 7: 0.93 ± 0.03) and MyH11 (day 2: 0.12 ± 0.001, *p* < 0.001; day 4: 0.20 ± 0.02, *p* < 0.05; and day 7: 0.30 ± 0.02). However, calponin (day 2: 0.25 ± 0.04, *p* < 0.001; day 4: 0.60 ± 0.02; and day 7: 0.56 ± 0.01) and smoothelin (day 2: 0.25 ± 0.02; day 4: 0.44 ± 0.03, *p* < 0.01; and day 7: 0.25 ± 0.01) showed the highest protein expressions on day 4 of culture.

## 4. Discussion

We hypothesized that SMC spheroids, with their better regenerative properties, could represent superior building blocks for future bladder TE projects. For the first time, we established and analyzed primary bladder SMC spheroids and compared them with traditional 2D cultures. We found that SMC spheroids were viable for up to 14 days. The main characteristic of smooth muscle is represented by the expression of important contractile proteins: *α*-SMA, calponin, smoothelin, and MyH11 [30]. Spheroids not only expressed the early contractile protein *α*-SMA; they predominantly expressed the late myogenic differentiation marker MyH11. In contrast, 2D SMC expressed higher quantities of the general SMC marker *α*-SMA and lower quantities of MyH11. The expression of ECM proteins in spheroids was markedly higher than in 2D SMCs. The excellent contractile ability of the SMC spheroids combined with higher ECM deposition is a promising factor for TE purposes, as functional SMCs and ECM deposition are a prerequisite for building a functional bladder wall substitute.

The remarkable advantages of spheroids, derived from other tissues for TE, have been reviewed extensively by other authors [20, 31, 32] and include improved viability, differentiation, function, ECM production, angiogenesis, and decreased inflammation response. Yet to our knowledge, this had never been explored with primary bladder-derived SMCs. We showed that a range of SMC seeding conditions form spheroids. The number of SMCs seeded correlated with the final spheroid size. The highest-seeded cell number yielded the largest spheroids. We did not observe any increase in spheroid size over time and therefore do not assume that SMCs proliferate within the spheroids. Instead, density increased, which is reflected in a decrease in the spheroid surface area during the observation period. This is in line with Jäger et al. [33], who were able to show that the assembly of SMCs in multilayered 3D culture conditions promoted cellular quiescence through downregulation of the expression of genes required for proliferation and protein biosynthesis while maintaining a process of cellular reorganization. They concluded that 3D cultures might generate cell aggregates with more stable SMC differentiation markers than conventional 2D cultures [33]. This is an important and beneficial finding, as SMCs are difficult cells to culture in traditional 2D cultures. Unfortunately, they lose their normal and contractile phenotypes at higher passages and tend to shift to a synthetic or proliferative phenotype that lacks contractile function [30]. A certain degree of dedifferentiation to the synthetic or proliferative SMC phenotype may be tolerable, but it is of utmost importance that tissue-engineered bladder transplants are functionally and structurally as similar as possible to the targeted bladder [8].

The SMCs in the spheroids remained viable throughout an entire week. Subsequently, we kept the SMC spheroids in stable culture for at least 10 days. However, after 2 weeks of culture, areas with dead SMCs developed in the center of the spheroids, as we had expected. In weeks 2 and 3, the majority of SMCs became necrotic. The development of central necrosis zones in spheroids is caused by undersupply of oxygen and nutrients. Larger spheroids are more prone to this, as the diffusion distance increases with diameter [34]. Our approach using primary bladder-derived SMC spheroids is novel, so no comparable literature is available regarding viability, not even for primary SMC spheroids from other tissue origins. It is clear that spheroids from cancer cells have different properties and therefore are not suitable for discussing our results [35].

Characterization of SMCs relies on specific marker proteins. As previously described in detail by our group, *α*-SMA is an early and unspecific SMC marker. It is often used to characterize preliminary SMC phenotype but can frequently be found in myofibroblasts and fibroblasts. Calponin, smoothelin, and MyH11 are distinct markers for mature, contractile, and functional SMCs [28, 30].

To quantify gene induction of contractile and ECM genes, we performed qRT-PCR. Interestingly, SMC spheroids showed some dynamic changes, with significant higher gene induction for smoothelin, calponin, and MyH11 in early spheroids on days 2 and 4 than on day 7. The 2D SMCs displayed significantly lower gene induction for all contractile genes without major changes over time. The same is applicable for the ECM. Early spheroids showed significantly higher gene induction for collagen III and fibronectin and in particular for elastin, which even demonstrated a 14-fold gene induction on day 2. In contrast, early 2D SMC showed significantly lower gene induction of all ECM genes. It seems conceivable that our observations of higher early spheroid gene induction result from the quiescence of gene induction that, as discussed previously, is due to progressing densification during spheroid formation. SMCs are much more active in the beginning of spheroid formation and enter a resting phase when spheroid formation and densification is completed [33]. In contrast, 2D SMCs seem to need some time to adapt to the environment after seeding: their activity surges, and gene induction increases steadily over the culture period.

SMC spheroids offer a stable SMC specific phenotype, as indicated by positive staining for contractile proteins *α*-SMA, calponin, smoothelin, and MyH11. Semiquantitative evaluation of the stained contractile proteins from a single plane confocal image of the spheroids was not feasible. Spheroids produced with our technique vary in size and shape; this could have led to false results when applying formulas assuming that the spheroids were completely round.

The picture is different with regard to quantification for ECM protein staining. Spheroids clearly show greater expression than 2D cultures. This seems unsurprising, because 2D SMCs adhere to the surface of a culture flask or dish. They do not rely on secreting a framework of ECM proteins to ensure their own structural integrity. SMC spheroids, cultivated on a nonadherent inverted pyramid surface, must behave differently. They are forced to interact with each other during their process of self-assembly [19]. This strengthens cell–cell interaction and secretion of supportive ECM proteins. The increased secretion of ECM proteins could be of great advantage for TE, because a physiological ECM environment stimulates intracellular signaling pathways and thus determines the fate and tissue-specific organization of cells [36]. In addition, the ECM influences a wide array of cellular processes, including migration, differentiation, and most importantly wound healing [37].

The contractile protein expression pattern is fundamentally different in the two culturing methods, which is in line with qRT-PCR results. SMC spheroids seem to differentiate relatively quickly. This is characterized by an early rise in and ongoing predominance of the late myogenic differentiation marker MyH11, which is only present in contractile mature SMCs, and an associated significant decline in the *α*-SMA fraction over time. However, 2D SMCs behaved in the opposite way, expressing significantly higher quantities of the early myogenic differentiation marker *α*-SMA.

Postconfluent SMCs in 2D cultures have been shown to produce higher MyH11 than subconfluent SMCs, suggesting that density-related growth arrest might promote cell differentiation [38]. In theory, and if observations of 2D cultures also apply to spheroid culture conditions, this mechanism could explain the protein expression pattern of SMC spheroids presented here. The increasing density of SMC spheroids over time might promote growth arrest, cellular quiescence, and cell maturation and may prevent SMC spheroids from shifting to a synthetic phenotype [33, 38].

Our results clearly show that SMCs behave fundamentally differently in spheroid and 2D cultures. Direct and quantitative comparison of the results of the two culture methods may therefore not be meaningful. Even though this study suggests advantages to using spheroids over 2D monolayer SMCs, further *in vivo* work is required to confirm these benefits on a functional level. Our future goal is to use the established SMC spheroids embedded in a compressed collagen scaffold; first, to analyze their contractile abilities *in vitro* and later to explore their advantages in regenerative potential and functionality in a rat bladder augmentation model.

## 5. Conclusion

Primary bladder SMC spheroids offer several benefits over 2D SMCs: they show more late contractile protein markers, indicating the higher state of maturation, and produce larger quantities of ECM proteins. We were able to confirm that SMC spheroids are promising building blocks for studying detrusor regeneration in detail and may provide improved function and regenerative potential, contributing to taking bladder TE a significant step forward.

## Data Availability

The data required to reproduce these findings is presented in this manuscript.

## Supplementary Materials

Supplementary Materials Figure S1: SMC spheroid formation. Full 7-day time-course of SMC spheroid formation for cell seeding conditions: 500, 1000, and 1500 SMCs per spheroid. Figure S2: Continued live-dead staining. Observation of SMC spheroid viability for 3 weeks. Propidium idodide (PI) uptake in the center of the spheroid starts increasing from day 10 with a peak in uptake at day 14. From day 14 onwards, cells start to become necrotic. Figure S5: immunoblotting. Full range of representative bands for spheroids and 2D SMCs for each time point.
