# Supplementary material for: Spheroids of Bladder Smooth Muscle Cells for Bladder Tissue Engineering
Source: Biomed Res Int. 2021 Nov 11;2021:9391575. doi: 10.1155/2021/9391575 (PMC8601859; doi:10.1155/2021/9391575)
Supplement: Supplementary Materials — Figure S1: SMC spheroid formation. Full 7-day time-course of SMC spheroid formation for cell seeding conditions: 500, 1000, and 1500 SMCs per spheroid. Figure S2: Continued live-dead staining. Observation of SMC spheroid viability for 3 weeks. Propidium idodide (PI) uptake in the center of the spheroid starts increasing from day 10 with a peak in uptake at day 14. From day 14 onwards, cells start to become necrotic. Figure S5: immunoblotting. Full range of representative bands for spheroids and 2D SMCs for each time point. [file 9391575.f1.docx]

**Supplementary data**

**Spheroids of bladder smooth muscle cells for bladder tissue engineering**

**
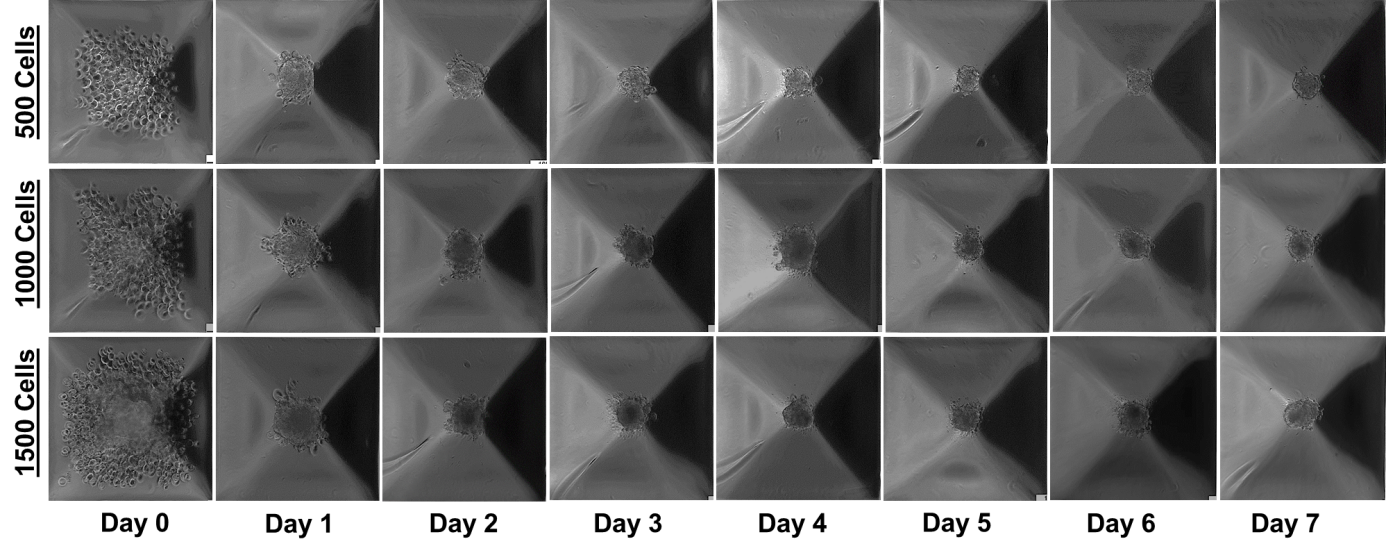
**

**Figure S1. SMC spheroid formation.** Full 7-day time-course of SMC spheroid formation for cell seeding conditions: 500, 1000, 1500 SMCs per spheroid.
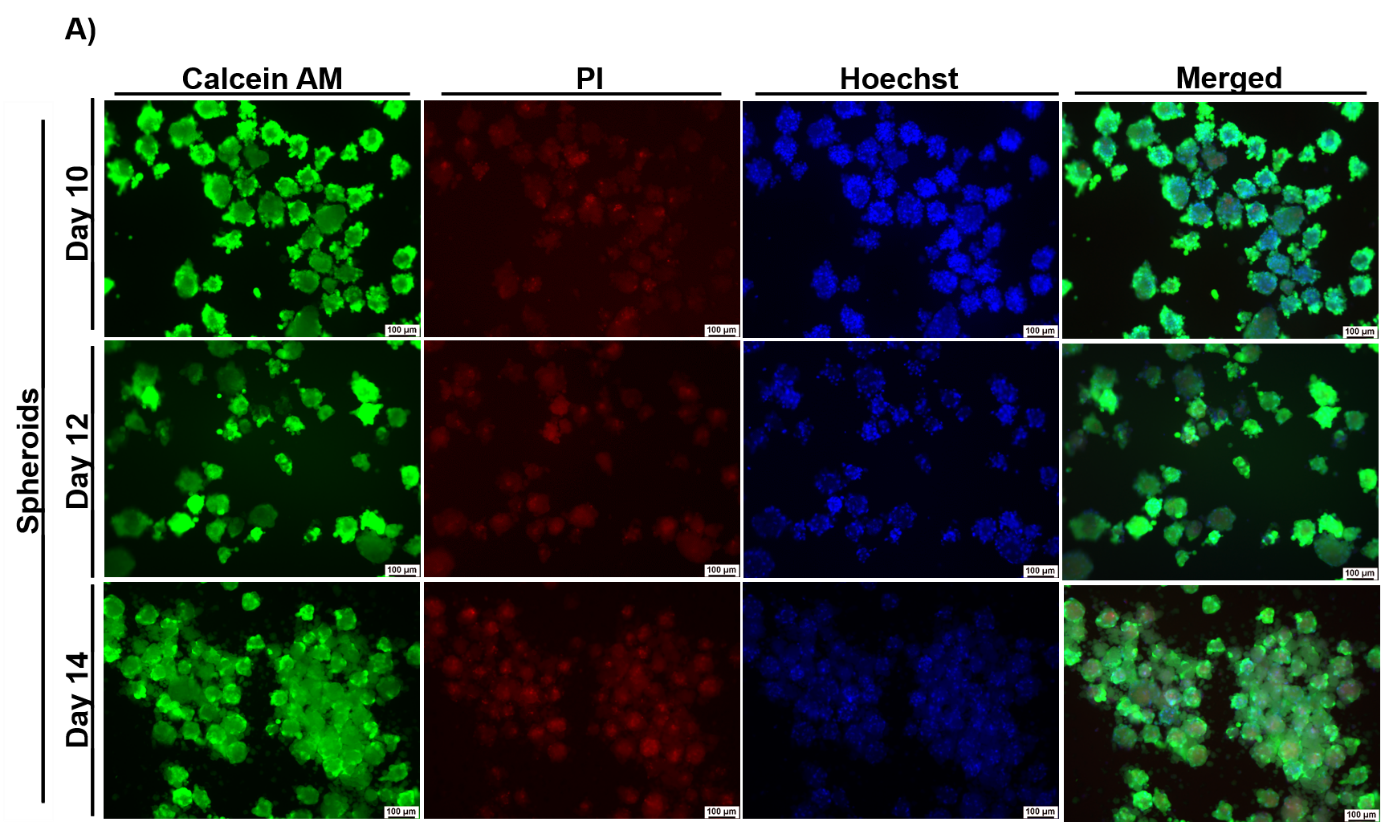


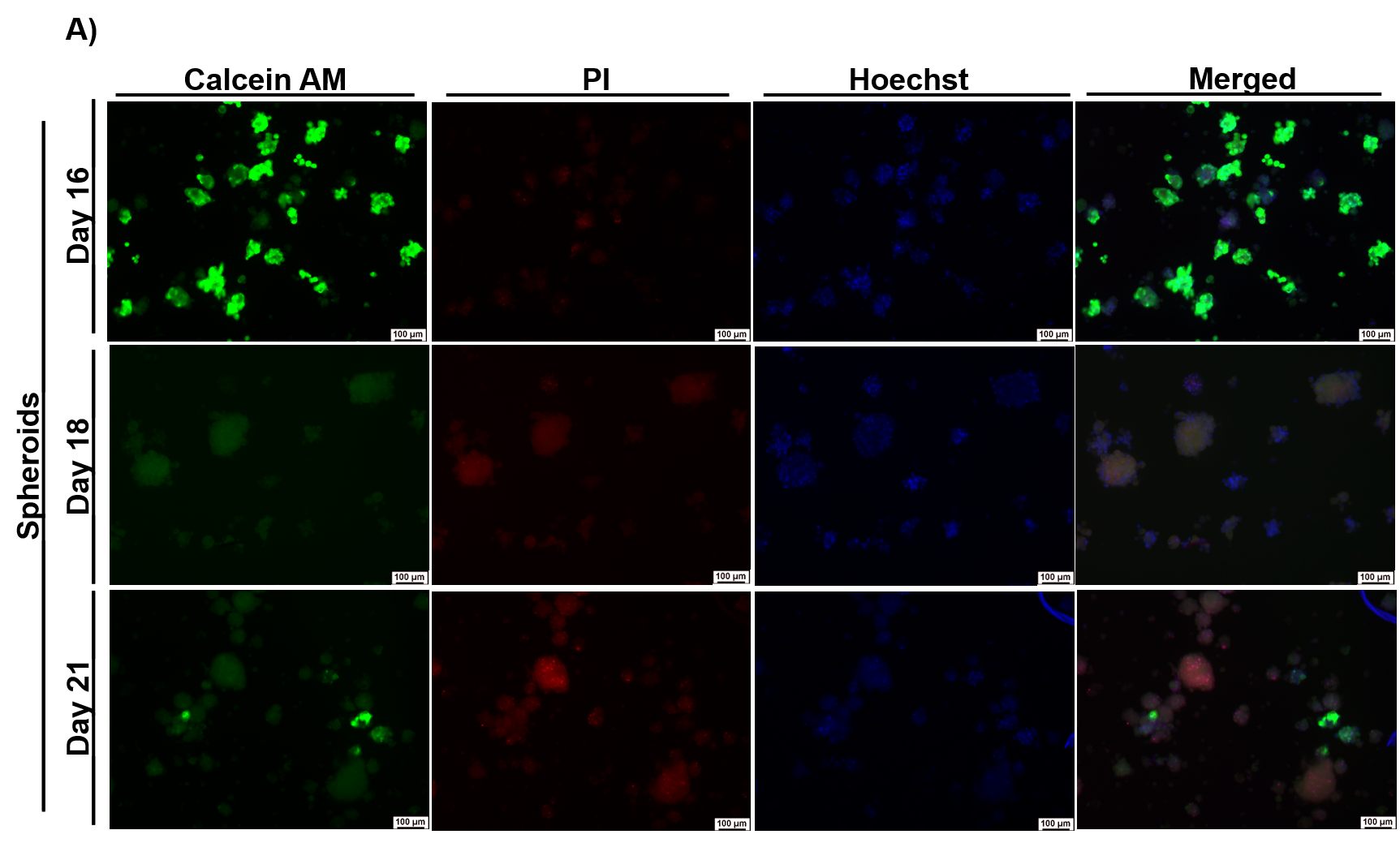


**Figure S2. Continued Live-Dead Staining.** Observation of SMC spheroid viability for 3 weeks. Propidium idodide (PI) uptake in the center of the spheroid starts increasing from day 10 with a peak in uptake at day 14. From Day 14 onwards, cells start to become necrotic.


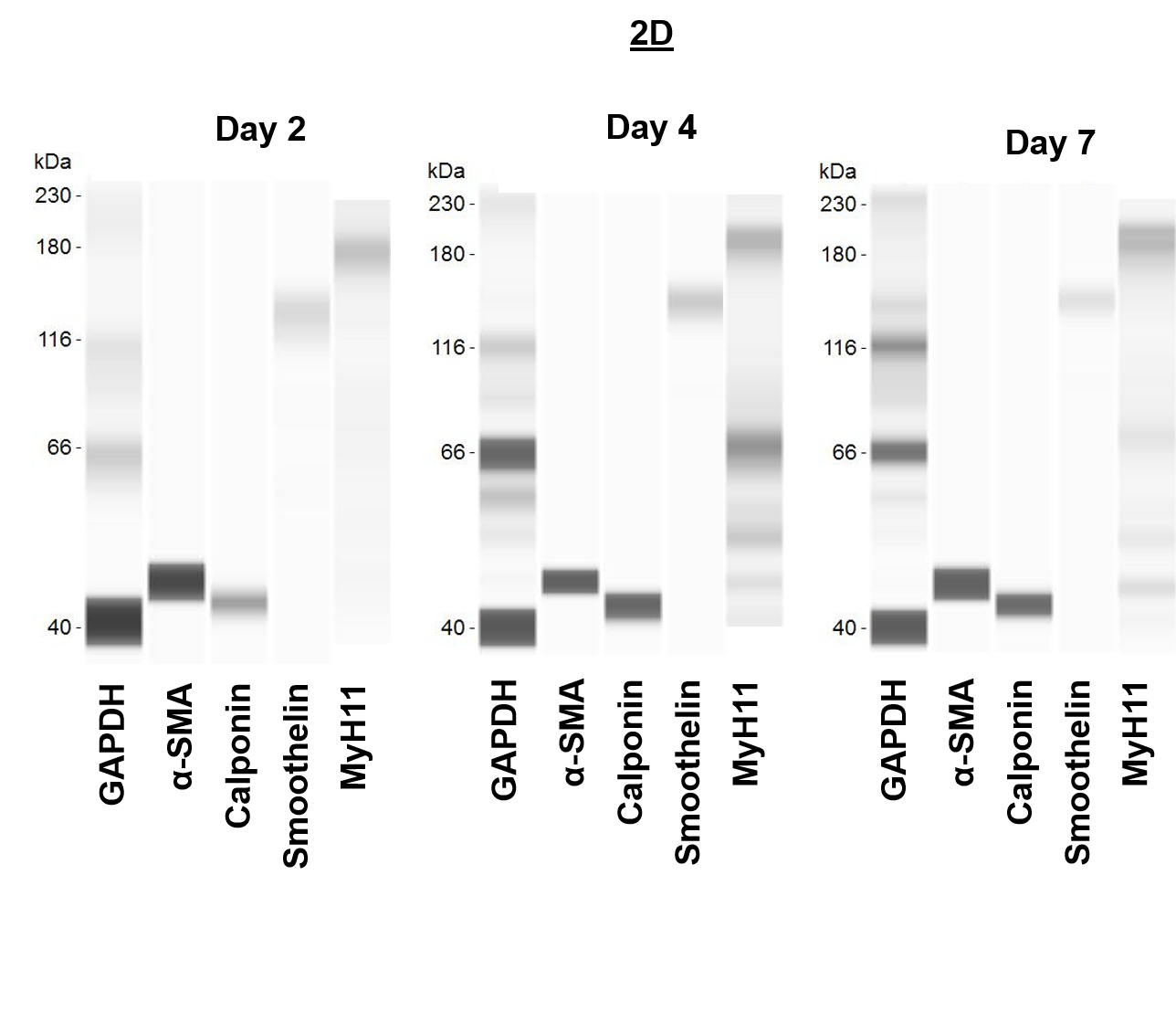


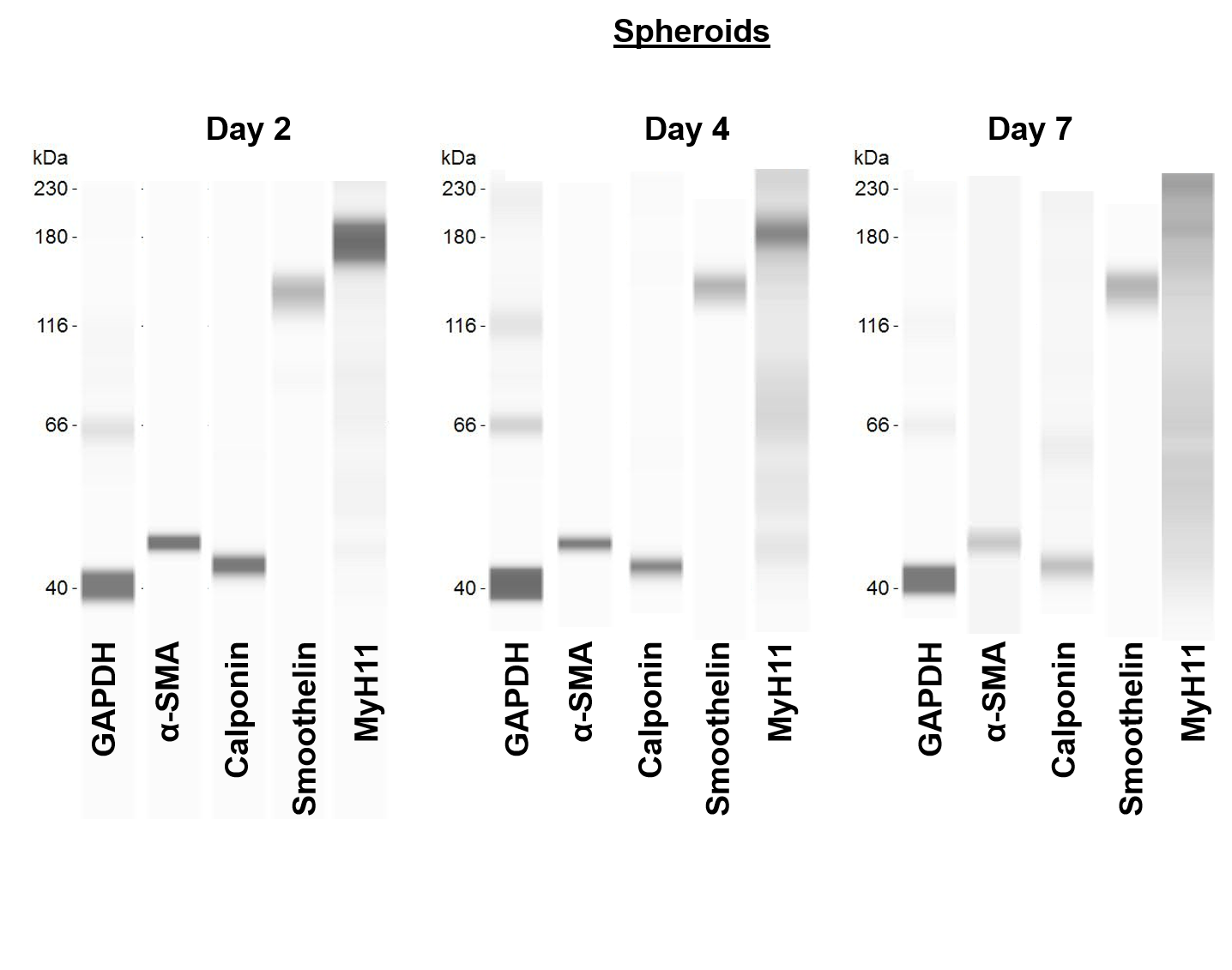


**Figure S5. Immunoblotting.** Full range of representative bands for spheroids and 2D SMCs for each time point.
